# Dancing on the platform: Lability of floral organs of *Beilschmiedia appendiculata* (Lauraceae)

**DOI:** 10.1002/ece3.8445

**Published:** 2021-12-07

**Authors:** Gang Zeng, Bing Liu, David K. Ferguson, Yong Yang

**Affiliations:** ^1^ College of Biology and Environment Nanjing Forestry University 159 Longpan Road Nanjing China; ^2^ Xishuangbanna Tropical Botanical Garden Institute of Botany Chinese Academy of Sciences Mengla China; ^3^ State Key Laboratory of Systematic and Evolutionary Botany Institute of Botany, the Chinese Academy of Sciences Beijing China; ^4^ Department of Paleontology University of Vienna Vienna Austria

**Keywords:** *Beilschmiedia*, evolution, flower, Lauraceae, ontogeny, phyllotaxis

## Abstract

Floral characters are important for the systematics of the Lauraceae. However, structure and development of the flowers remain poorly known in the family. In this study, we observed the variation and early development of flowers of *Beilschmiedia appendiculata*, which belongs to the *Cryptocarya* clade of the family. The results indicate that the shoot apical meristems (SAMs) of the floral buds are enlarged and become a platform for the programmed initiation of the floral organs; floral organs develop basically in an acropetal pattern; phyllotaxis is whorled, initiation of floral primordia within a whorl is asynchronous; floral merosity is extremely variable, for example, dimerous, trimerous, tetramerous, dimerous plus trimerous, and trimerous plus tetramerous. In addition, this species has lost the innermost staminal whorl and glands are not closely associated with stamens of the third staminal whorl, which is unusual in the family Lauraceae. Our new observations broaden our knowledge of the variation of floral structure in *Beilschmiedia* and pose a fundamental question regarding the ecology underlying the lability of floral organs in *B*. *appendiculata*.

## Graphical abstract

Floral merosity of *Beilschmiedia appendiculata* is extremely variable, for example, dimerous, trimerous, tetramerous, dimerous plus trimerous, and trimerous plus tetramerous. This species has lost the innermost staminal whorl, and glands are not closely associated with stamens of the third staminal whorl, which is unusual in the family Lauraceae.

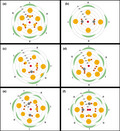

## 1 INTRODUCTION

The Lauraceae are trees or shrubs with the exception of the herbaceous vine *Cassytha* L. and have a pantropical distribution in Tropical America, Asia, Australia, and Africa (Kostermans, 1957; Rohwer, 1993). This family consists of ca. 55 genera and 2500–3500 species with two diversity centers in Tropical America and Tropical Asia (Rohwer, 1993; van der Werff & Richter, 1996). Many trees of this family are dominant in tropical and subtropical forests in both the Old World and the New World (Kostermans, 1957; Rohwer, 1993).

In contrast to their prominent habit and their vast distribution, these trees possess rather small flowers which bear taxonomic significance but are poorly known because of the difficulties of field collection and anatomy of the herbarium specimens (Yang et al., 2012; Zeng et al., 2017). This prevents the clarification of the taxonomy and a better understanding of the evolution in the Lauraceae. The *Beilschmiedia* Nees group is such a case in point.

The *Beilschmiedia* group is monophyletic and sister to the genus *Cryptocarya* R. Br. (Li et al., 2020; Rohwer et al., 2014; Song et al., 2020). This group consists of ca. 400 species which are classified into a few closely related genera, for example, *Beilschmiedia*, *Endiandra* R. Br. (Syn.: *Brassiodendron* C.K. Allen and *Triadodaphne* Kosterm.), *Hexapora* Hook. f., *Potameia* Thouars, *Sinopora* J. Li et al., *Syndiclis* Hook. f., and *Yasunia* van der Werff (van der Werff & Nishida, 2010; Yang et al., 2012). Generic delimitation within this group has been difficult due to the lack of morphological observations, low divergence of DNA sequences, and poor sampling of molecular systematics (Liu, 2013; Rohwer et al., 2014; Yang et al., 2012).

Floral merosity and organ number have frequently been used in generic delimitation of the *Beilschmiedia* group. *Brassiodendron* C.K. Allen has trimerous flowers possessing 6, two‐locular stamens and was considered to be a heterogeneous group with some species ascribed to *Beilschmiedia*, while other species should be classified under *Endiandra* (Hyland, 1989; van der Werff, 2001). *Triadodaphne* has trimerous flowers with 3 two‐locular stamens and strongly unequal tepals and was either treated as a synonym of *Endiandra* or as a separate genus (Hyland, 1989; van der Werff, 2001). Both *Sinopora* and *Hexapora* have trimerous flowers with six stamens (Li et al., 2008; van der Werff, 2001). Species of the genus *Syndiclis* have both dimerous and trimerous flowers possessing variable numbers of stamens ranging from 4 to 9 (Zeng et al., 2017). Unlike *Syndiclis*, the Malagasy *Potameia* has dimerous flowers lacking the staminodes of the fourth whorl (van der Werff, 1996). In *Beilschmiedia*, the flowers are mostly trimerous, but dimerous flowers do occur (van der Werff, 1996).

Allen (1942) established a monospecific genus *Lauromerrillia* C.K. Allen including only *L*. *appendiculata* C.K. Allen and indicated that this genus is similar to *Beilschmiedia* but differs from the latter by the trimerous flower having six stamens. Lee and Wei (1982) dissected flowers of the paratype specimen *S*.*K*. *Lau 3535* and found that *L*. *appendiculata* actually possesses flowers with stamen number ranging from 6 to 8, suggesting that stamen number in the two genera varies continuously. As a result, they reduced *Lauromerrillia* to synonymy under *Beilschmiedia* and made a combination [*B*. *appendiculata* (C.K. Allen) S.K. Lee & Y.T. Wei]. Due to the difficulty of dissecting the small flowers, some aspects of the variation of floral characters remain unclear: (1) Is the continuous variation an incorrect observation or a real case? and (2) what is the structural variation of the flowers?.

In this study, we investigated the early development and anatomy of flowers of *B*. *appendiculata* using both light microscopy and scanning electron microscopy to reveal morphological and structural variation of floral organs in the species and discuss its implications within a phylogenetic context.

## 2 MATERIALS AND METHODS

**FIGURE 1:**
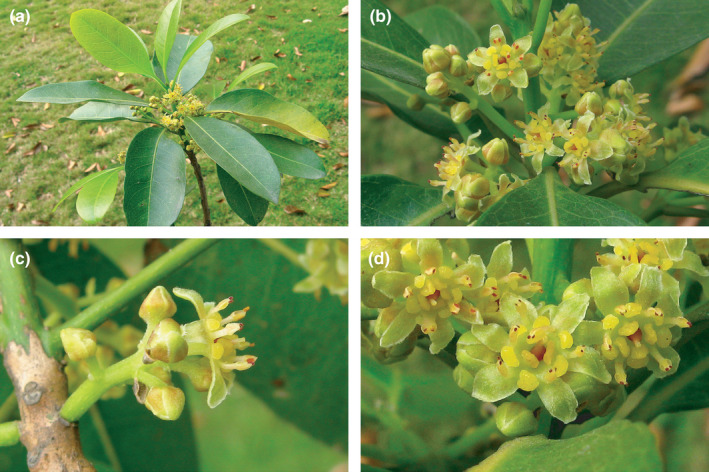
Macromorphology of *Beilschmiedia appendiculata*. (a) A floral branch; (b) magnification of (a) showing inflorescences; (c) lateral view of a flower; (d) apical view of flowers

Floral materials of *B*. *appendiculata* were collected in the South China Botanical Garden (Figure 1a–d). Young inflorescences with different developmental stages were collected by Yi‐Hua Tong on March 2, 15, 22, and 28 of 2014. Plant materials were fixed in FAA (mixture of Formalin, Alcohol, and Glacial Acetic Acid) in the field. The materials were then immersed in new FAA liquids and permanently deposited in the laboratory. Pickled materials and voucher specimens of the species were deposited in the Herbarium (PE), State Key Laboratory of Systematic and Evolutionary Botany, the Chinese Academy of Sciences, and Herbarium (NF), Nanjing Forestry University.

Anatomy and ontogeny were studied using both light microscopy and scanning electron microscopy. For anatomical studies, floral materials were immersed in alcohol (75%), and then, they were observed and dissected to expose the primordia of floral organs under a light microscope (LM). Those flowers with different developmental stages and metamorphic variation were selected for further observations. The selected materials were then dehydrated in an ethanol series, critical point dried, coated with gold palladium, and fixed to stubs. The material on the stubs was observed and photographed in a HITACHI S‐4800 scanning electron microscope (SEM) at 10.0 kV at the State Key Laboratory of Systematic and Evolutionary Botany, Institute of Botany, the Chinese Academy of Sciences.

For microtome sections, the dehydrated materials were transferred to xylene and embedded in paraffin. Successive sections were made using a rotary microtome (4 µm thick). The sections were then placed on microscope slides and stained with safranin‐fast green. We observed the sections and took micrographs under a Leica DM 5000 B light microscope. Images were edited and adjusted using Adobe Photoshop CS2 vers. 9.0.

## 3 RESULTS

In the South China Botanical Garden, *B*. *appendiculata* initiated its inflorescence buds in late November, followed by a long stasis, as flower buds with early developmental primordia of tepals were not found until March 2. The plant started to bloom around the end of March and continued flowering until May.

Flowers were assembled to determinate thyrsoid panicles, peduncles ended with one or rarely two flowers at the apex. Internodes of the paniculate inflorescences became increasingly shorter toward the distal end. Distal flowers of the inflorescences appeared to be subopposite. The proximal internodes of the inflorescences were relatively longer, and flowers appeared to be alternate. Development of the inflorescences was basipetal: the apical flowers opened first, while the proximal flowers bloomed later.

Development of the flower buds was relatively synchronous, these flower buds appear similar in morphology and prominence. Early in the initiation of the flower buds, the shoot apical meristem (SAM) was enlarged due to rapid peripheral growth, the SAMs were usually nearly round or fusiform. Basically, the flowers developed in an acropetal manner, that is, primordia of the outermost whorl first, followed by the inner whorls developed centripetally.

**FIGURE 2:**
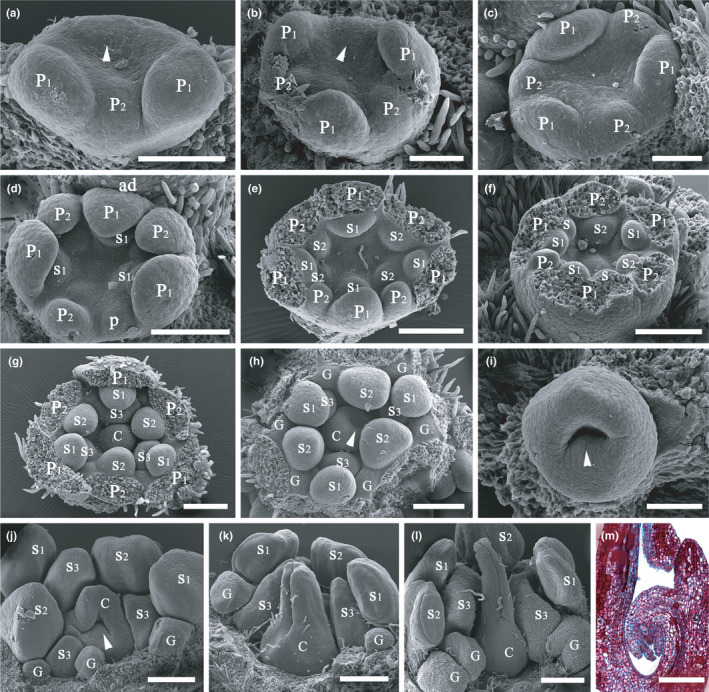
Development of flowers of *Beilschmiedia appendiculata*. (a) Early development of a trimerous flower, the three primordia of the outer tepal whorl (P_1_) with different prominence, arrow showing the position of a developing tepal primordium whose initiation is later than the tepal primordia of the inner tepal whorl; (b) a trimerous flower bud with initiation of all three tepals of the outer whorl, beginning of development of the inner tepal whorl, but the tepal primordia having different developmental rates, arrow showing the position of an expanding tepal primordium; (c) a trimerous flower bud with two whorls of well initiated tepal primordia; (d) a metamorphic flower bud with initiation of three staminal primordia (S_1_), one additional tepal primordium (P) occurring near a tepal of the inner whorl, the adaxial primordium more prominent than the abaxial primordium; (e) a tetramerous flower bud, the four primordia of the first whorl of stamens (S_1_) initiated, two upper tepal primordia more prominent than the lower two tepal primordia (P_2_), the staminal primordia corresponding to the larger tepal primordia in the same row bigger than the staminal primordia corresponding to the smaller tepal primordia; (f) a trimerous flower bud, the left‐side tepal primordium of the inner tepal whorl less prominent, two additional staminal primordia (S) occurring near two staminal primordia (S_1_), both axillary to the outer whorl of tepals (P_1_); (g) a regular trimerous flower, primordia of the third whorl of staminodes initiated, carpel primordium (C) also initiated; (h) a regular trimerous flower bud, glands initiated, arrow pointing to concavity of the apical‐lateral side of the carpel primordium; (i) the carpel primordium, arrow marking the position of a forthcoming ovule in the cross‐zone; (j) a regular trimerous flower bud, arrow indicating the ovular protuberance, the two flanks of the carpel converging on one another from apical portion downwards to the cross‐zone, the three primordia of the third staminal whorl having very different developmental rates; (k) the carpel with almost closed ventral suture; (l) further fusion of the ventral suture, the upper portion of the carpel elongated into style, the filaments prolonged; (m) a longitudinal section of the ovary displaying the two integuments of the anatropous ovule. Scale bars: a–c, and i: bar = 100 µm; e–h, j, and m: bar = 200 µm; k: bar = 300 µm; l: bar = 500 µm. ad, adaxial; C, carpel; G, glands; P, tepals of irregular occurrence; P_1_, the outer whorl of tepals; P_2_, the inner whorl of tepals; S, staminal organs with irregular position; S_1_, the first/outermost whorl of stamens; S_2_, the second whorl of stamens; S_3_, the third staminal whorl including either fertile stamens or staminodes; S_4_, the fourth staminal whorl including staminodes

The three or four spaced tepal primordia of the outer whorl (P_1_) were initiated first at the edge of the SAMs, but their initiations were asynchronous (Figure 2a). Soon, the tepal primordia of the second whorl (P_2_) started to develop between the primordia of the first whorl (P_1_). The primordia of the second whorl were also asynchronous. Sometimes, a primordium of the first whorl was initiated somewhat later than the primordium of the second whorl, leading to a more prominent primordium of the second whorl than the primordium of the first whorl (Figure 2a). Normally, the enlarged primordia of the first whorl were slightly broader than the primordia of the second whorl. In a normal flower, the primordia of the first whorl were equal in number to the primordia of the second whorl, and the six tepal primordia were decussate (Figure 2b,c). However, sometimes there were two primordia instead of one primordium (Figure 2d).

The primordia of the third whorl (S_1_) were initiated in line with the primordia of the first whorl (P_1_) and alternate to the primordia of the second whorl (P_2_) (Figure 2d). For the lateral flowers of a cyme, the adaxial primordia were usually initiated earlier than the abaxial ones, which led to the adaxial primordia being more prominent than the abaxial primordia (Figure 2d). The primordia of the fourth whorl (S_2_) parallel to the primordia of the second whorl (P_2_) were alternate to the primordia of the third whorl (S_1_) (Figure 2e). Timing of the primordial initiation of the fourth whorl (S_2_) appears to be related to the parallel primordium of the second whorl (P_2_) in the same row; that is, if one primordium of the second whorl (P_2_) was initiated earlier, then the corresponding primordium of the fourth whorl (S_2_) was also initiated earlier (Figure 2e). Normally, the primordia of the first and the third whorls were equal in number, but sometimes, there were two primordia of the third whorl corresponding to one primordium of the first whorl (Figure 2f). The primordia of the third and the fourth whorls developed further into fertile stamens. The primordia of the fifth whorl (S_3_), corresponding to those of the third whorl (S_1_), were alternate to those of the fourth whorl (S_2_) (Figure 2g,h); these primordia normally develop into staminodes. There were only three staminal whorls in *B*. *appendiculata*, and the fourth staminal whorl is never initiated.

In a normal flower bud, the pistil consists of a single carpel (C) (Figure 2g–j). The carpel primordium developed almost at the same time as the primordia of the staminodes in the fifth whorl (S_3_). Initially, the central portion of the floral bud formed a protuberance which was dome‐like and had no clear differentiation into growth zones (Figure 2g). Then, the upper to lateral surface of the dome became indented (Figure 2h), and the carpel flanks and the lower cross‐zone became visible. The ovular primordium was initiated at the cross‐zone, that is, the middle inner portion between the flanks (Figure 2i). With further development of the carpel primordium, the ovule primordium increasingly curved inwards and downwards, and an anatropous ovule was finally formed (Figure 2m). The carpel flanks approximated each other distally, the enclosure proceeded downwards and finally the carpel completely enclosed the ovule (Figure 2k). At this time, the filaments began to elongate and pubescence started to develop on the filaments, carpel, and tepals. Then, the upper portion of the carpel was prolonged to form a narrow style (Figure 2l). The style possessed a longitudinal groove at this stage.

Glands of the staminodes first appeared after the carpel primordium started to become indented (Figure 2g,h). The glands were sessile and irregularly globose close to maturity, while the epidermal cells became swollen and granular. The epidermal cells of the third whorl of staminodes (S_3_) changed in a similar way (Figure 2k,l).

Normally, this species has flowers possessing two whorls of tepals, three staminal whorls, and one central pistil. The outer two whorls of stamens (S_1_ and S_2_) were introrse and fertile; the inner third whorl (S_3_) was latrorse and usually sterile, but occasionally fertile (Figure 2j). The fourth whorl of staminodes normally occurring in most Lauraceae was absent in this species.

**FIGURE 3:**
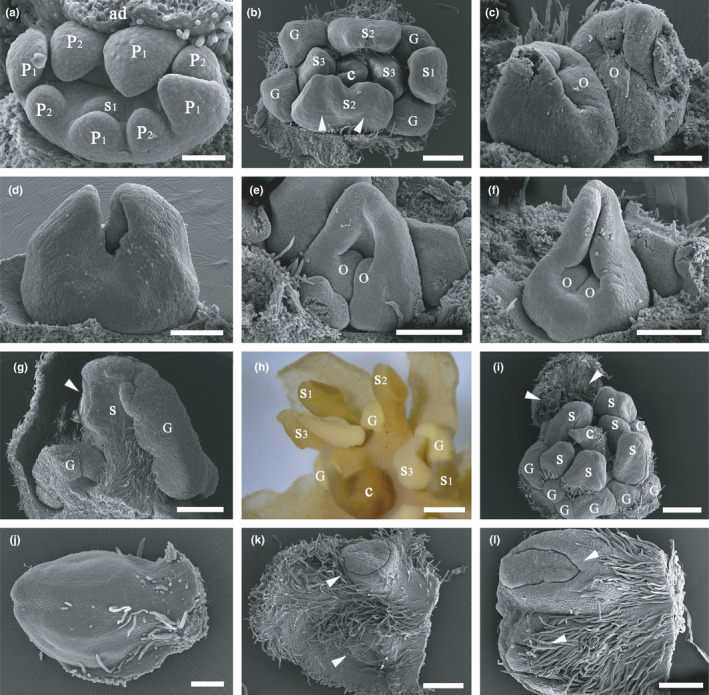
Metamorphic development in *Beilschmiedia appendiculata*. (a) A tetramerous flower bud showing the more prominent adaxial primordium; (b) a dimerous flower bud, arrow pointing to two fused anthers of a stamen in the second staminal whorl (S_2_); (c) two carpels face‐to‐face on the ventral side, each carpel bearing an ovule (O); (d) two opposite carpels connate at the base; (e) a young carpel bearing two ovular protuberances, the ventral suture down to the base of the carpel; (f) a carpel bearing two ovular protuberances at the cross‐zone; (g) a fertile stamen fused to a lateral gland (G), arrow indicating an anther cell; (h) a fertile stamen (S_3_) fused to a gland (G); (i) glands with irregular position and size, arrows pointing to the anther cells of a chimera of tepal and stamen; (j) a chimera of stamen and tepal, similar to a stamen; (k) a chimera of stamen and tepal, similar to a tepal, arrow indicating the anther cell; (l) a chimera of stamen and bract from the basal portion of an inflorescence, arrow showing the flap of a well‐developed anther cell. Scale bars: a and c: bar = 100 µm; d–f, j and l: bar = 200 µm; b and k: bar = 300 µm; g and i: bar = 500 µm; h: bar = 1 mm. ad, adaxial; C, carpel; G, glands; O, ovule; P_1_, the outer whorl of tepals; P_2_, the inner whorl of tepals; S, staminal organs with irregular position; S_1_, the first/outermost whorl of stamens; S_2_, the second whorl of stamens; S_3_, the third staminal whorl including either fertile stamens or staminodes; S_4_, the fourth staminal whorl including staminodes

**FIGURE 4:**
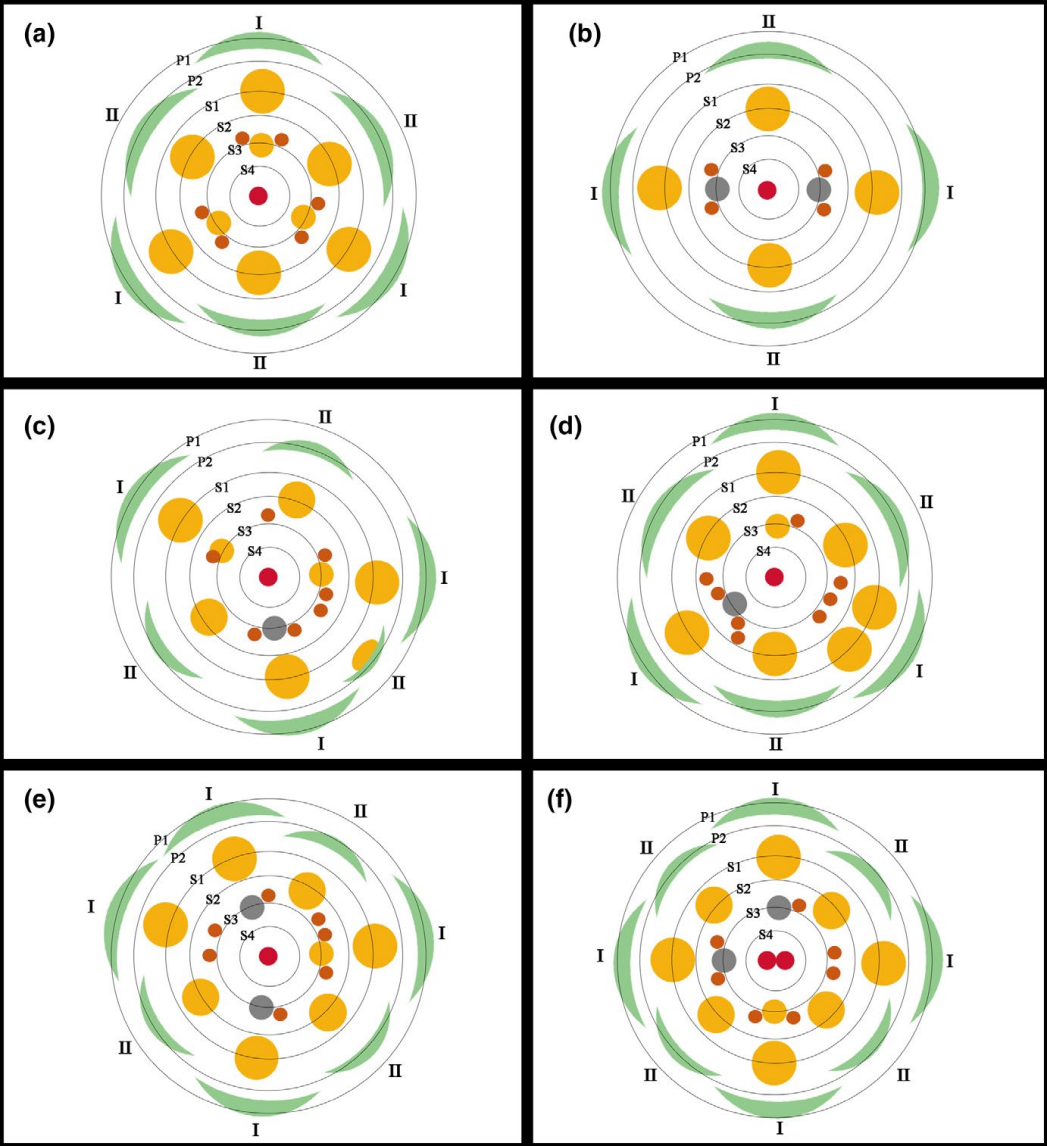
Illustrations displaying variation of floral organs of *Beilschmiedia appendiculata*. (a) A trimerous flower displaying the regular Lauraceous floral structure; (b) a dimerous flower displaying the regular arrangement of floral organs and glands, the third whorl of stamens becoming staminodes; (c) a mosaic flower of tri‐ and di‐merosity displaying the fertile stamens of the third whorl, glands irregularly arranged, mosaics of stamens and glands, and mosaics of tepals and stamens; (d) a trimerous flower displaying the stamens of the third staminal whorl becoming staminodes or missing, glands irregularly arranged, two stamens sometimes in place of one stamen; (e) a mosaic flower of tetra‐ and tri‐merosity displaying the stamens of the third staminal whorl becoming staminodes or missing, glands sometimes absent; (f) a tetramerous flower displaying the stamens of the third staminal whorl becoming staminodes or missing, glands sometimes absent, the pistil consisting of two carpels. Green crescents refer to tepals; gray solid circles indicate staminodes, yellow solid circles are fertile stamens, orange circles are glands, and red circles are pistils. P_1_, the outer whorl of tepals; P_2_, the inner whorl of tepals; S_1_, the first/outermost whorl of stamens; S_2_, the second whorl of stamens; S_3_, the third staminal whorl including either fertile stamens or staminodes; S_4_, the fourth staminal whorl including staminodes

The number of tepals, stamens, glands, and position of the glands varied greatly in the species. The number of tepals and stamens varied from 6 to 8, rarely 4 or 5 (Figure 3a,b). We counted 126 flowers and found that trimerous flowers are the most frequent merosity (54.8%, Table 1). The number of glands was usually variable, and these glands were often irregularly inserted on the disk of the nearby fertile stamens. Occasionally, the pistil possessed two carpels which were opposite at the ventral suture (Figure 3c,d). Within the unicarpellate pistil, there were sometimes two ovules (Figure 3e,f). Additional metamorphic patterns were present in this species. Sometimes two stamens were fused (Figure 3b). Occasionally, the glands were fused to fertile stamens of the third whorl (S_3_) (Figure 3g,h). In other cases, a fertile stamen and a tepal formed a chimera (Figure 3i,j,k). Some of the chimeras were similar to tepals (Figure 3i,k), some to stamens (Figure 3j). These chimeras only occurred at the position of tepals, never at the staminal positions (Figure 3i). In addition, these chimeras were usually smaller than a normal tepal. These chimeras seem to develop from the smaller primordia in the first and second whorl of tepals (P_1_ and P_2_) (Figure 2e,f). Sometimes, there was also a chimera of an anther and a basal bract of an inflorescence (Figure 3l). Variation of floral organs of *B*. *appendiculata* is summarized as illustrations (Figure 4a–f).

**TABLE 1:** Frequencies of flowers of *Beilschmiedia appendiculata* with different tepals

Tepals of a flower	Flowers	Ratio (%)
Eight	10	7.94
Seven	14	11.11
Six	69	54.76
Five	27	21.43
Four	6	4.76

## 4 DISCUSSION

### 4.1 Asynchronous initiation of floral organs in a whorl

Floral phyllotaxis is considered to be an important aspect in understanding the evolution of flowers (Endress & Doyle, 2007). Endress and Doyle (2007) and Endress (2011) defined floral phyllotaxis by two parameters, that is, divergence angle of two successively initiated organs with regard to the center of a flower, and plastochron between the initiation of two successive organs. According to them, all organs in a spiral flower are initiated sequentially in more or less equal plastochrons and with roughly equal divergence angles along the ontogenetic spiral, while the organs in a whorled flower appear in pulses with unequal plastochrons and unequal divergence angles. The flowers of the Lauraceae are whorled according to this definition; that is, the plastochrons and divergence angles change dramatically between whorls but are relatively equal or nearly so within a whorl. The flowers of *B*. *appendiculata* are seemingly whorled, but floral organs are initiated asynchronously within a whorl (Figure 2a,b,d). For a lateral flower, the adaxial primordium is usually initiated earlier and is more prominent than the abaxial primordium (Figure 2d). This developmental pattern seems common in the Lauraceae (Buzgo et al., 2007; Kasapligil, 1951; Singh & Singh, 1985; Zeng et al., 2017). The floral phyllotaxis of *Endiandra montana* appears to be spiral (Hyland, 1989). Further studies are necessary in additional taxa of the family to better understand the details. The Lauraceae are primitive angiosperms, which may account for the asynchronous initiation of floral organs in a whorl.

### 4.2 Tepals and androtepals

Tepals are sterile foliar organs surrounding the inner fertile elements, and there are usually six tepals arranged in two whorls in the Lauraceae (Rohwer, 1993). Different opinions exist regarding the origin of tepals. Buzgo et al. (2007) suggested that the tepals in the Lauraceae are derived from the abortion of stamens. Cronquist (1988) believed that the tepals in archaic angiosperms are modified leaves. Eames (1961) contended that the perianth had a dual origin in angiosperms, the inner whorl (i.e., petals) being derived from sterilization of stamens while the outer whorl (i.e., sepals) was modified from leaves.

According to our observations in this study, the tepals appear homologous to stamens. They are initiated in a similar series as foliar appendages on a condensed floral shoot, and there are gradual transitions between tepals and stamens, sometimes chimeras bear features of stamens and tepals in the position of tepals. In addition, the first staminal whorl is transformed into a third whorl of tepals in *Dicypellium* and *Phyllostemonodaphne*, and the two outer staminal whorls are tepaloid (Rohwer, 1993). Tepals are sometimes also transformed into stamens or completely reduced in *Litsea* and *Lindera* (Rohwer, 1993). Hyland (1989) reported that tepals developed into anthers or vice versa in *Endiandra montana*. In this study, we observed morphological transitions between tepals and stamens in *B*. *appendiculata*. The tepals of the perianth whorls are frequently modified into stamen‐like chimeras (Figure 3i,j,k), suggesting tepals are reduced stamens.

Developmental genetic studies provide insights into the evolution of flower organ identities. Based on comparisons of the transcriptional patterns in *Persea* and *Arabidopsis*, Chanderbali et al. (2009) support a fading borders model of floral organ identity in basal angiosperms (Soltis et al., 2007). According to this hypothesis, C function is primary in carpel development and B function is dominant in perianth development, B and C class organ identity genes jointly control the development of male gymnosperm cones, the bisexual angiosperm flowers with apical female and lower male originate from ancestral male gymnosperm cones by reduction of the B function in the distal region of male gymnosperm cones, while subsequent reduction of the C function at the proximal region leads to the derivation of sterile perianth organs. This hypothesis explains the gradual changes in floral organs in basal angiosperms. It does work as an explanation for some metamorphic patterns in the *Beilschmiedia* group.

### 4.3 Staminodes

Typical flowers of the family Lauraceae normally possess four different kinds of floral organs in a flower, that is, tepals in two whorls, fertile stamens in three whorls, staminodes in one whorl, and a central unicarpellate pistil. Our observations support the concept that the staminodes in the Lauraceae are reduced stamens with the major portion of the stamen reduced followed by fusion of lateral glands (Buzgo et al., 2007). In *B*. *appendiculata*, the staminode whorl in a normal Lauraceous flower has been lost and fertile stamens of the third staminal whorl are frequently found to be replaced by staminodes. Intermediate morphology between glands and stamens is frequently found in the third whorl of fertile stamens (Figure 3g,h), and epidermal cells of the glands and the secretory portion of the staminodes show similar morphological changes, both are granular before maturity. Sometimes fertile stamens of the third whorl become staminodes in *B*. *appendiculata*. This phenomenon was also observed in *Syndiclis* (Zeng et al., 2017). It seems that fertility of the innermost staminal organs increases the diversity of flowers. However, it remains difficult to understand the underlying genetic network that regulates the development of the staminodes using only the fading borders model of Chanderbali et al. (2009). Probably the amount of expression of the B function plays a role in regulating the formation of staminodes.

### 4.4 Carpel number of the pistil

Different opinions exist as to how many carpels the pistil of the Lauraceae contains (Buzgo et al., 2007; Cronquist, 1981; Eames, 1961; Feng, 1963; Kostermans, 1957; Sastri, 1965; Wang, 1995; Wang et al., 2000). Some contend that the pistil consists of a single carpel, that is, unicarpellate (Buzgo et al., 2007; Kasapligil, 1951; Rohwer, 1993). Others are inclined to believe that the pistil resulted from a reduction or fusion of three carpels (e.g., Kostermans, 1957; Li, 1982; Reece, 1939; Singh & Singh, 1985), thus pseudomonomerous in the family. Pseudomonomery is defined as a gynoecium consisting of seemingly only one carpel but actually representing several carpels merged and partially reduced. Vascular anatomy has been considered to be conservative and provides indications of vestiges of reduced or fused organs. The ovary in the Lauraceae possesses six vascular bundles, which was taken as evidence that the ovary in the Lauraceae was derived by fusion of multiple carpels, which was rejected by Rohwer (1993).

According to our observations in this study, the ovary of *B*. *appendiculata* normally consists of only a single carpel which is comparable to the free carpels in other primitive angiosperms, but rarely two free or basally fused carpels replace the unicarpellate pistil. Bi‐ or tri‐carpellate status was also observed in *Cassytha*, *Phoebe*, *Sassafras*, *Syndiclis*, and *Umbellularia* (Feng, 1963; Kasapligil, 1951; Wang, 1995; Zeng et al., 2017). Previous authors hypothesized that the unicarpellate ovary, which is normal in the Lauraceae, was probably derived by reduction of a pluricarpellate pistil (Cronquist, 1981; Wang et al., 2000). It is well known that the gynoecium possesses one carpel per flower in the Hernandiaceae and Lauraceae, but is pluricarpellate in other families of the Laurales (Staedler, 2008). The unicarpellate gynoecium of Lauraceae is probably derived, because the basal family in the Laurales is pluricarpellate (Massoni et al., 2014; Renner, 2011).

### 4.5 Number and position of ovules in the pistil

The pistil of the Lauraceae has been described as containing a single anatropous ovule (e.g., Kasapligil, 1951; Rohwer, 1993; Zeng et al., 2017). In this study, we observed a certain variability. There are sometimes two ovular primordia in the ascidiate carpel in *B*. *appendiculata* (Figure 3e,f). Within the Laurales, a carpel having two ovules is only found in the Calycanthaceae (Endress & Igersheim, 1997; Renner, 2004). The family Calycanthaceae represents the basalmost branch within the Laurales (Massoni et al., 2014; Renner, 2004). As a result, we consider that the two‐ovuled carpel in *B*. *appendiculata* is a kind of reversal to the ancestral state. The one‐ovuled carpel is a result of reduction in the two ovule states in the common ancestor of the core Laurales. Abortion of one of the two ovules was observed in the Calycanthaceae (Endress & Igersheim, 1997). The position of the single ovule is related to the number of ovules in the single carpel of the pistil in Lauraceae. The ovule primordium is normally at the upper rim of the cross‐zone of the ascidiate carpel when there is only one ovule, but the cross‐zone is divided into two symmetrical parts and the ovules become almost contiguous with (the proximal and adaxial side of) the carpel flanks when there are two ovules in the carpel (Figure 3e,f).

### 4.6 Unusual floral characters of *B. appendiculata*

*Beilschmiedia appendiculata* was first described as a separate genus *Lauromerrillia* based on the flower having six stamens (Allen, 1942). Lee and Wei (1982) noticed that it has a variable number of fertile stamens: six or occasionally eight stamens, and thus incorporated *Lauromerrillia* into *Beilschmiedia*. We confirmed the observations of these earlier authors but provide more details. *Beilschmiedia appendiculata* does possess unusual flowers that are either trimerous or occasionally tetramerous, and the outer two staminal whorls are fertile whereas the third staminal whorl is sometimes partially fertile resulting in the variation of stamen number of the species. A similar situation also occurs in *Syndiclis* aff. *malipoensis* (Zeng et al., 2017). However, our detailed study shows that the flower of *B*. *appendiculata* lacks the fourth staminal whorl which is usually present in other species as staminodes. Floral organs are reduced in miniature flowers of the *Beilschmiedia* group, but it is unusual to have reduced floral organs in a relatively large‐flowered species as in *B*. *appendiculata*. Despite the unusual features, *B*. *appendiculata* clearly belongs to the core *Beilschmiedia* group according to a recent phylogeny (Li et al., 2020).

Reduction of stamens is parallel in the *Beilschmiedia* group and derived multiple times in the *Beilschmiedia* group because the six‐stamened species are distributed in several clades according to recent phylogenetic studies (Li et al., 2020; Rohwer et al., 2014; Zeng et al., 2017). In China, there are two species of *Beilschmiedia* having six‐stamened flowers. Besides *B*. *appendiculata*, *B*. *pauciflora* H.W. Li from southern Yunnan also possesses flowers with six fertile stamens (Lee & Wei, 1982). The 2 six‐stamened species together with the nine‐stamened *B*. *delicata* and *B*. *tsangii* constitute a clade which receives low to moderate support (Li, 2020). Due to the lack of a robust phylogeny, it remains unclear whether the reduction of stamens of the two Chinese species resulted from a common ancestor or arose independently.

The presence and modification of glands have increased the diversity of flowers of the Lauraceae. In a typical flower of the family, each stamen of the third whorl normally possesses two glands at the base of the filament. In *Brassiodendron*, *Chlorocardium*, *Phyllostemonodaphne*, *Urbanodendron*, all fertile stamens are found to bear glands at the base of the filaments (Rohwer, 1993). In *Pleurothyrium*, the glands are distinctly enlarged surrounding the base of all stamens (Rohwer, 1993). In some species of *Endiandra*, the glands are concentrated into a glandular cushion (Hyland, 1989). In *Anaueria*, *Hexapora*, and *Williamodendron* and a few species of *Endiandra*, *Licaria*, and *Mezilaurus*, the glands are completely lacking (Rohwer, 1993). The Chinese *Syndiclis* species were considered to have glands associated with stamens of the first whorl (Li, 1982). However, a recent developmental study has shown that the glands are actually associated with stamens of the third whorl (Zeng et al., 2017). In *B*. *appendiculata*, the number and position of the glands were found to be variable (Figure 4c–f), the glands are irregularly inserted on the disk of the nearby fertile stamens but not associated with the stamens of the third whorl, and sometimes, the glands are fused to fertile stamens of the third whorl, which is unusual in the Lauraceae.

Flowers are an important component of diversified reproductive systems, and floral morphological diversity of angiosperms is closely related to animal pollination (Campbell & Powers, 2015; Ramos & Schiestl, 2019). It is evident that pollinators have an intense influence on floral traits (Gómez et al., 2014; Rusman et al., 2019). Differentiation of pollinator visitation has been found between quaternary and quinary flowers in *Ruta graveolens* (Tang & Ren, 2011). As a result, it is reasonable to infer that the lability of floral merosity in *Beilschmiedia appendiculata* might have been caused by changes in selective pressure from pollinators though we have no direct evidence. Bisexual flowers of the Lauraceae are protogynous. For a typical heterodichogamous flower of the family (*Persea americana*), the flower opens twice and the floral phenology includes two different phases (Rohwer, 2009: *Machilus grijsii*; personal observation). In the first phase (the female phase), all the stamens are curved outwards and spreading when the flower is open, anther cells remain closed but the staminodes are showy and produce abundant nectar at this time, while the glands are dry and not showy (Rohwer, 2009). The flower closes after a few hours of the female phase. In the second phase (male phase), when the pistil is pollinated, the third whorl of stamens moves from spreading to become upright and surround the central pistil. The anther cells are open now, but the staminodes are withered, while the glands become moist and showy and secrete nectar (Rohwer, 2009). Finally, both stamens and tepals become contiguous distally and the flower appears closed (personal observation). In *B*. *appendiculata*, the flowers usually have six stamens belonging to the outer two staminal whorls, whereas stamens of the third whorl are frequently/partially specialized as staminodes. It seems unnecessary to have both glands and staminodes functioning at different times. These structural changes may be related to functional shifts of the reproductive system in *B*. *appendiculata*, but it is unclear how the floral organs cooperate in six‐stamened species such as *B. appendiculata*.

## CONFLICT OF INTEREST

The authors declare that there is no conflict of interest.

## AUTHOR CONTRIBUTIONS

**Gang Zeng:** Data curation (equal); Formal analysis (equal); Investigation (lead); Methodology (lead); Writing – original draft (supporting). **Bing Liu:** Conceptualization (supporting); Formal analysis (supporting); Funding acquisition (supporting); Investigation (supporting); Resources (lead). **David K. Ferguson:** Writing – review & editing (supporting). **Yong Yang:** Conceptualization (lead); Formal analysis (lead); Funding acquisition (lead); Investigation (supporting); Methodology (supporting); Project administration (lead); Supervision (lead); Writing – original draft (lead); Writing – review & editing (lead).

## Data Availability

All data used in the study are included in this paper.
